# Is One Health a Viable Strategy in Animal Health Litigation: Evidence from Civil Lawsuits in China

**DOI:** 10.3390/ani11092560

**Published:** 2021-08-31

**Authors:** Kai Wu, Ying Yu, Chen Chen, Zheming Fu

**Affiliations:** 1 School of Law, Zhongnan University of Economics and Law, Wuhan 430073, China; wukai@zuel.edu.cn; 2 School of Public Administration, Zhongnan University of Economics and Law, Wuhan 430073, China; 3 School of Health Sciences, Wuhan University, Wuhan 430071, China; chenchen835@pku.edu.cn; 4 School of Law, Peking University, Beijing 100871, China; zhemingfu@pku.edu.cn; 5 Maurer School of Law, Indiana University Bloomington, Bloomington, IN 47405-7000, USA

**Keywords:** animal law, litigation strategy, One Health, law and economics, strategic litigation

## Abstract

**Simple Summary:**

Strategic litigation launched to protect animal welfare worldwide branches out with several tactical themes: environmental protection, child abuse, veterinarian malpractice, product liability and quasi-family member. Currently, the litigation strategy themed in One Health has been observed in legal practice in Chinese mainland. Using 1520 zoonosis related civil lawsuit judgments, this study aimed to assess the effectiveness of this litigation strategy in animal health cases from Chinese mainland. It has been confirmed that using the litigation strategy themed in One Health results in more successful outcomes and larger damage awards, so there might be a practical value in using this strategy in animal welfare lawsuits.

**Abstract:**

Several litigation strategies are used to gain support from courts in order to protect animals. While the emerging litigation strategy themed in One Health stimulates judicial protection in the animal health sector, little is known about whether and how such strategies are supported by courts. In this article, we investigate how animal welfare litigation strategies influence judge’s choices within their discretion. We argue that litigators equipped with the litigation strategy themed in One Health are placed in an advantageous position in animal health cases, but that this tendency varies markedly across zoonoses. Specifically, we suggest that litigators utilizing One Health’s litigation strategy are associated with higher probabilities to win, whereas normal litigators are not. Further, we propose that litigators equipped with the One Health litigation strategy are awarded more damages from judges. We test and find support for our predictions using a cross sectional dataset of civil lawsuit cases centering on the animal health industry in Chinese mainland. Our findings indicate that courts indeed were persuaded by the One Health litigation strategy, even when bound by the discretion rules. At the same time, we suggest that for advocates who would like to litigate for animal welfare in the animal health sector, the litigation strategy themed in One Health might have potentially positive implications.

## 1. Introduction

Animal protection needs effective strategies. Animal welfare advocates are continuously trying to do their best to protect animals while minimizing the potential threats and risks. They will consider seeking help from the legal system when they perceived clear signals of the danger. For instance, between 1 January 2014 and 31 December 2018, 4735 cases involving wildlife protection were reported in China [1]. These lawsuits are always time-consuming, costly, and exhausting. Instinct tells us that launching such kind of lawsuits is worthwhile for we see can strategic litigations here and there in the courtroom to create broader and proper changes in society [2,3,4,5,6] and animal welfare advocates need to disseminate their willingness thus inspire more people to protect animals [7,8,9]. However, there might be other messages sent to the public by these animal protection lawsuits: animal abuse exists, and this infringement is so serious that judicial action is required. At the end, how judges decide those cases reflects the strength of the judicial protection for animal welfare in a particular country or region. There are two concerns embedded in this support: When faced with various forms of animal protection litigation, how do judges react and how they write their thoughts into court decisions? To obtain a more advantageous position in future litigations and to better protect animals, how can animal welfare advocates who filed the case optimize the ways and methods they litigate in light of prior landmark court judgments?

Although a tremendous number of animal protection cases can be collected through legal research, the existing knowledge around them still cannot provide a clear answer for the concerns above. Current studies about animal welfare focus on statutes and the appropriate penalty; their common thread is an attempt to relate the issues with ethics, customs, and family values [10,11]. For instance, as Calley [12] claimed, the statutes that underpinning animal welfare law provide a lot of discretion for ambiguity and subjective interpretation. Rubin [13] suggested that a well-designed approach that includes punishment, deterrence, rehabilitation, restoration, and protection for both human and nonhuman animal victims are needed. Although the current studies clarified the legal implications of animal welfare breaches, research on the court strategies connected to animal welfare protection is still scarce (or more specifically research on whether the animal welfare protection court strategies in China is effective). For the animal health sector which is closely linked with animal welfare [14], researches remain vacant in China.

As a result, one of the objectives of this paper is to deliberate the evolution of animal welfare litigation strategies, as well as their respective features. The use of historical review is the first attempt in the literature to illustrate the animal welfare protection “toolbox’,’ which includes multiple litigation strategies, while also providing a framework to scientifically analyze how the court responds to these strategies. The second goal of this paper is to assess the court’s response by filling another gap in the literature. The first question addressed here is whether the most recent lawsuit tactic (dubbed the One Health approach) is advantageous to participators of animal health litigations. In addition, the court’s responses have two sides of impact in general. The first is the reputation side, which refers that the court’s proclamation of victory or defeat bolstered the reputation of animal health advocates. The second is the monetary side, which means that the court’s judgement of the amount of damage provided material assistance to animal health advocates. As a result, it begs the second question: in what sense is the latest litigation strategic approach effective?

The courts’ attitude toward strategic litigation in patent, antitrust, and environmental protection cases have previously been thoroughly researched in the field of law and economics [15]. Literature shows that some courts ignore this type of litigation, while others have preliminarily recognized its positive importance. However, we only identified one study conducted by Song in 2020 in the animal welfare protection area that discussed the importance of strategic litigation in animal protection [16]. His study employed a total of 1360 animal-related lawsuits that were litigated in the United States from 1865 to 2010, with the emphasis of summarizing the strategic activities in animal litigation. As a result, he categorized 35 key issues to illustrate how litigants might attain positive outcomes by strategic actions. Nevertheless, Song’s study is limited in that the conclusions are hazy, focusing exclusively on how plaintiffs’ strategic activities may lead to the formation of new regulatory agencies. Based on this gap we identified, we believe that there is a need for a more comprehensive study with practical implications in the interdisciplinary area of law and animal welfare protection.

Using a total of 1520 cases filed by federal and local courts in Chinese mainland, we studied how courts in Chinese mainland responded to instances involving zoonotic diseases. Considering that courts in China are classified into four levels: the Supreme one, the higher ones, the intermediate ones, and the primary ones (the district ones) and each court’s jurisdiction over specified cases fits the territorial boundary. Additionally, there are also specialized courts in China dealing with only one type of cases, such as maritime, intellectual property, finance, railway transport, and cyberspace affairs. All these courts receive large quantities of case everyday. In some of these cases, the One Health strategy, which has recently emerged in China [17,18,19,20,21,22], was employed. Our paper generates two practical implications which enriched the existing literature. First, China built a public and comprehensive online database that provides all accessible case details to stakeholders (including judges, litigants, attorneys, and others) in order to assist them in defending themselves in court [23]. There are over 25,000 pieces of judgments in China being uploaded into this database every day. This allows us to retrieve information on the regularly occurring animal protection cases and provides a large sample size across a range of areas, including companion animal, agricultural animal, laboratory animal and wildlife. A pattern and the effectiveness of the litigation strategy can be concluded and utilize in the future animal protection cases. Second, China, being the world’s biggest animal trader and the home of the world’s largest agricultural industries [24], may be viewed as a pioneer in experimenting with cutting-edge strategies for animal welfare protection. Since the animal protectionists in other countries have comparative tools (or incentives) to preserve animal welfare, the adoption of these strategies in China can benefit the entire globe. Although the legal system varies across countries, and some of the variables adopted in this study may be exclusive to China, the theory and litigation strategy proposed in this study can be applied to other countries and regions with animal protection laws.

Our study also makes two theoretical contributions. First, we categorized and assessed all known animal welfare-related litigation strategies, resulting in a categorization guide for future study in this field; second, based on the judicial discretion theory and legal strategy theory, we investigated the reaction of all level courts in Chinese mainland to the so called One Health litigation strategy (or litigation strategy themed in One Health), and assessed the efficacy of this very unique litigation strategy in animal health lawsuits.

We present the first thorough empirical study of the real impact of pursuing better outcomes in animal health lawsuits through litigation strategies in Chinese courts. The empirical findings of our study will help animal health advocates evaluating their present litigation strategies, rationally adopting the emerging One Health litigation strategy in the future, and even considering optimizing this unique litigation strategy in other countries. It is because our results indicate that litigants who adopted the One Health litigation strategy perceived more support from courts and judges. This support is evident in the fact that litigants who participate in animal health lawsuits via One Health litigation strategy have a higher possibility to win and a higher amount of damage payment. Furthermore, another reason is that our findings also reveal that judges showed cautious support for the One Health concept, which is viewed as a public policy goal, within the extent of their discretion. Such kinds of support may be ubiquitous and widespread.

The paper organized as follows: Section 2 examines the existing litigation strategies worldwide, as well as their application in China. Section 3 explains the methodology used, followed by a thorough section on the main findings, which is limited in a Chinese background. Then the study finalized with an in-depth discussion of the study’s findings in the context of China and their generalizability in Section 4, Section 5 and Section 6 concludes.

## 2. How Animal Welfare Is Protected in Courts: Different Types of Litigation Strategies

There is no national animal welfare law enacted in China at present, and the legal protection for animal welfare varies among different animal categories [25]. For instance, laboratory animal welfare was the first among all animal categories to be protected by related statutes [26]. Meanwhile, the legislation and regulations for farm animals are still in the early stages and developing. For domestic animals, despite the lack of specific regulation on animal welfare for them, pets have traditionally been culturally recognized as part of family possessions [27], which means they might be protected through the the real property section in Chinese Civil Code (formerly the real right law). As a response to COVID-19, China’s Congress immediately approved a ban on the consumption of wildlife [28]. However, in terms of public awareness and reality, the perceptions of animal welfare protection by the public are ahead of the judicial system [29,30,31], although some judges in China wrote their concerns about animals into judgment. For example, in a civil case (*Yefang Qian v. Xiaofei Zhu*) where the plaintiff sought to give homeless cats a better shelter [32], the judge from a Suzhou court wrote that:

- … …Each homeless animal that was rescued needs much care from the rescuer. The Court haw to point out that adopters of homeless animals, such as the defendant, should strengthen their awareness of duty by devoting their affection, being careful in their breeding duties, and being sincere in their adoption promises….

Started with the judicial reform since 2010 in China, symbolic cases with high quality judgments have the potential to be selected as Guiding Case, which is quite similar to the precedents in case law in western countries. All these facts mentioned above leave space for the use of litigation strategies in the sphere of animal welfare protection, which we shall discuss later.

The concept of strategic litigation originated from the legal strategy theory [33]. The essence of this theory is that there are strategies to infuse new meaning into the blank spaces of the law in court debates for legal dominion that is still under development or has altered significantly [33]. The strategic litigation can be filed based on social norms, community expectations, and so-called “public policies”. When writing judgments for those cases, the judge may be persuaded and endorse the plaintiffs’ viewpoint. In practice, mature litigation strategies have evolved in the domains of human rights, patent protection, environmental protection, and women’s rights protection already [34,35] with landmark cases from China [36,37].

In order to bring suit, the plaintiff must have a “cause of action”, which usually consists of harm caused by the defendant for which the law provides a remedy [38]. Animal welfare advocates must have a comprehensive grasp of numerous possible animal welfare breaches, and abilities to formulate litigation strategies based on the varied features of these violations, thus develop and establish successful animal welfare protection litigation strategies. In this study, we analyzed and categorized the current animal welfare protection related litigation strategies, by evaluating the textual content of approximately 1000 animal cases acquired from animal law literature. Apart from the data obtained from the cases, we also conducted a thorough assessment of the literature on strategic litigation theories to ensure that no important categories were overlooked. As a consequence, five mature litigation strategies were identified: filing suits under environmental laws; filing suits on legal basis of child abuse; filing suits on legal basis of vet malpractice; filing suits on legal basis of product liability; and filing suits under family laws.

Table 1 lists the definitions and representative examples of each litigation strategy. There are significant variations between these five types of strategies, as can be shown. Some strategies can only be utilized in certain circumstances. The strategy of filing lawsuits under family law, for example, is mostly used in domestic animal cases, while the use of product liability law to protect animal welfare can be extended to laboratory animals and farm animals. Furthermore, some litigation strategies may be used to very specific animal categories, such as launching cases under environmental regulations, which can be applied to zoo animals. Furthermore, some litigation strategies may be used to specific animal categories, such as launching cases under environmental regulations, which can be applied to zoo animals.

### 2.1. An Overview of Current Animal Welfare Protection-Related Litigation Strategies

Litigation strategies appeared to be a means to persuade courts, and they have already gained popularity in the domain of animal welfare protection. In practice, not all legal strategies allow the animal welfare advocates to effectively accomplish their expectations, although it is reasonable for them to have high expectations especially for the case they filed as an animal welfare lawsuit. The reason is that the use of this category of litigation strategy requires full consideration of both the content of the law and the existence of judge’s judicial discretion.

First, the judicial discretion when making judgments provides space for the existence of litigation strategies. It empowers a judge to make decisions based on his or her own distinctive evaluation, governed primarily by legal principles [47]. It also bestowed enormous authority on courts, which is deployed only when the legislative permits it. Unlike the traditional perception of judges in China, they are not as mechanical as vending machines now. The freedom for judges to objectively evaluate a case with his or her own perception is considerably larger in emerging domains where the law is not well formed, such as in animal law, than in areas of criminal law with a precise and comprehensive rule system. The existence of this freedom in judgment making does not affect a country’s authority of judicial system and judicial branches, rather, it is a manifestation of the art of judicial adjudication.

In addition, scholars have developed numerous litigation strategy theories to explain which litigation strategy is the most appropriate one in the context of law. The theory we adopt in this study regards the law was delivered as the mixture of intricate interactions among written laws, law in lawyers’ minds, social norms, law in use, system imperatives, and outcome expectations. Under this theoretical framework, prior research in other developed legal domains has provided numerous resources for the formation of animal welfare protection litigation strategy.

After an extensive search of the literature and publicized court records, we concluded that there are five litigation strategies that were widely adopted in prior animal welfare protection related cases. If the legal system for animal welfare protection is not mature enough, particularly in developing countries, these strategies tend to start from other angles, such as environmental protection, child protection, veterinary malpractice, product liability, and family members. The details of these litigation strategies were deliberated Table 1 along with the response from the court system.

#### 2.1.1. Environmental Protection

Since environmental law has a longer history in comparison with animal law, the statutes involving animal protection were usually can be found directly in almost every country’s environmental law before the animal law system was shaped [48]. This worldwide legal consensus creates favorable conditions for incorporating environmental law into animal protection. There are two components to this sort of legal strategy. On the one hand, animals are considered as organic components of the ecosystem, and improving the general environment necessitates improving animal welfare in the ecological context as well [49]; on the other hand, this strategy is mostly adopted at the micro level, and it varies case by case. For instance, a lawsuit can be filed to urge cattle and poultry farms to stop releasing contaminants that impair animal welfare [50].

#### 2.1.2. Child Abuse

This litigation strategy originates from the scientific discovery of the association between animal cruelty and domestic violence [51]. It has been proven that children who witness or suffer from animal cruelty or neglect are more likely to mimic the behavior of the perpetrator and get involved with animals and human violence in the future [52]. If it is verified that the experience of witness animal cruelty will nurture the potential for domestic violence in the future, a plausible strategy for minimizing domestic violence is lowering the frequency of animal cruelty scenarios might be conceivably observed by children. This strategy has dual effects, which lie in the restrictions on animal cruelty and the protection of animal welfare. Below is one paragraph from the judgment made by a Jingzhou court (*Qin Li v. Yuanqing Fan*) [32] which clearly shows the judge’s attitude towards animal cruelty.

- …Cat abuse and the use of vlogs to show the suffering a cat has gone through is clearly a lack of compassion and respect for life, and they are against human morality and social civilization. The use of the internet by any individual or organization should adhere to public order and good morals, respect social morality, and avoid any violence, blood, cruelty, or other information that may cause physical and mental discomfort. Openness, sociality, and pervasiveness characterize cyberspace. Cat abuse or even selling cat abuse videos on social media should be discarded and banned, since these behaviors not only mislead people who are unable to properly recognize social phenomena, but also go against the core socialist values, are detrimental to the formation of a decent social order, and violate good customs….

#### 2.1.3. Veterinarian Malpractice

Veterinarians comprise the prevention, diagnosis and treatment of animal disease or injure. They will inevitably face with the needs of both the animals and the animal owners during this process. As a result, their malpractices or not providing appropriate care and treatment may result in not only a loss of animal welfare, but also lawsuits launched by animal owners, with allegations such as negligence, bailee responsibilities, breach of animal medical contract obligations, and deceptive trade practice [53] (See *Chaopeng Zhong v. Shanghai Duoqu Companion Animal Co. Ltd.* [43]).

#### 2.1.4. Product Liability

The primary legal basis of this litigation strategy is the connection between food safety and animal welfare protection. The Chinese public was first drawn to farm animal welfare by concerns over meat safety and food security has been the top priority of the state’s food security strategy [54]. Animal welfare policies have been proven to be a viable tactic for guaranteeing high-quality food production [55]. For instance, Lannetti [56] claimed in his study that the adoption of strict animal welfare standards on the ranch and during transportation can improve the microbiological safety of poultry meat. The litigation strategy, which focuses on whether the product satisfies the standards, has the power to compel poultry product manufacturers to enhance animal welfare (See *Hongmei Chen v. Dongning County Dianna Russian Commodity Shop* [45]), which is quite similar to the mechanism used in food and drug public interest litigation. This sort of lawsuit tactic may readily grab the public’s attention, especially because China has now experienced COVID-19, and currently places a premium on food safety and human health [57].

#### 2.1.5. Quasi-Family Member

The emotional connection between humans and animals is essential to the litigators who launch lawsuits from the perspective of family law [58,59]. It is has been argued that part of the legal obligations for properly treating family members is manifested in the principles and provisions of family law, which can be applied to animals that have formed a mutual companionship relationship with humans, particularly companion animals, to promote their well-being [60]. Despite the fact that this perspective has not yet been popularized throughout the world, Chinese courts have begun to acknowledge the unique status of companion animals (See *Jun Li v. Shanghai Peishi Animal Clinic Co. Ltd.* [41]). The recognition of the emotional bond between humans and animals, on the other hand, is largely geographical in nature, and is more common in major cities with more pets where people keep cats and dogs not for catching mice, guarding homes or other working purposes but as companion or family members [61].

### 2.2. The Courts’ Response to Strategic Litigations

**Figure 1:**
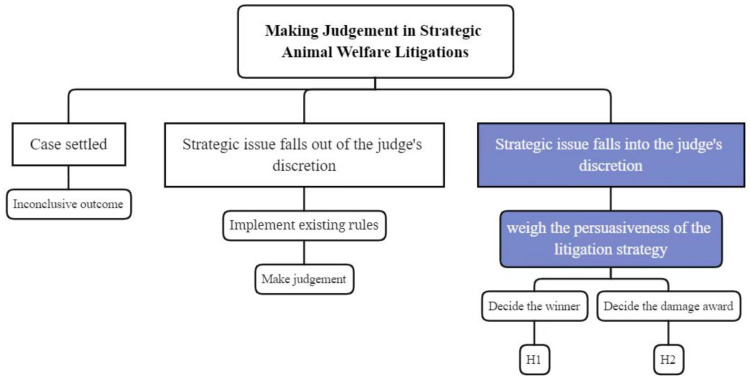
The ways judges react to litigation strategies.

In fact, although there are sorts of litigation strategies that have been employed to protect animal welfare, not all of them have yielded the expected results. To begin with, the degree of support from Chinese courts varies depending on the animal welfare litigation strategy. For instance, only one case involving child abuse has been supported by a Chinese court (*Qin Li v. Yuanqing Fan* mentioned above), whereas many cases utilizing environmental protection litigation strategies have been supported. Second, judges’ attitudes differ even in cases where the identical litigation tactic was employed, confirming the presence of the aforementioned judicial discretion. In family law cases, for example, some courts acknowledge that the inherent characteristics of companion dogs distinguish them from the property in the broadest and common sense. Pet dogs are living beings, and all living beings should be appreciated. Man’s harmony with nature, and man’s harmony with animals, are symbols and prerequisites of modern civilized civilization. To some extent, pet dogs can interact and converse with people. Their capacity to interact and communicate with humans allows them to establish spiritual bonds and emotional reliance with humans. Despite the fact that these courts acknowledge the special status of companion dogs, they continue to deny compensation claims for mental harm caused by the loss of animal life. Meanwhile, some courts have acknowledged that the loss of pets has resulted in some mental harm since pet owners have spent greater emotional and financial resources in the parenting process. As a result, they have backed pet owners’ requests and factored emotional value into the compensation packages (See *Yue Tian & Yun Zhao v. Zigong Hightech Zone Yichong Pet Garden* [62]). The mind map of how judges react to litigation strategies was shown in Figure 1.

### 2.3. The Emerging Litigation Strategy Themed in One Health in Animal Health Lawsuits

Since the scenarios involving animal welfare in the modern world are constantly changing and quite complicated, existing litigation strategies are sometimes unable to meet animal welfare advocates’ expectations. Rather than passively maintaining the *status quo*, the animal protectionists are actively pioneering with new strategies based on the changing environment around the courts. They constantly track the changes in national public policies concerning animal welfare, public expectations, and the boundaries at what the courts can or will tolerate, then create the appropriate courtroom moments for new litigation strategies. In fact, we have observed comparable trends in the evolution of strategic litigation in other domains, such as intellectual property rights protection and environmental protection [63,64,65,66]. In this study, we notice and observe that a new litigation strategy themed in One Health is frequently used in civil lawsuit cases involving zoonotic diseases tactically.

Generally the analytical framework of One Health stresses the reciprocal effect of human, animal, and environmental health, with the goal of achieving overall health for all participants [67,68]. It also highlights the essential role of environmental agents in the process of disease transmission, emphasizing the importance of multidisciplinary, inter-departmental, and inter-regional collaboration [69]. International organizations such as the WHO [70], OIE [71], FAO [72], and the World Bank [73] have already placed a high value on the application of this analytical framework, which has already shown promise in addressing global public health and poverty alleviation issues [74,75,76].

Though researchers are trying to define the scope of One Health and then use this term, there is still no consensus on what constitutes One Health precisely up to now. However, the absence of a precise definition does not undermine the strategic value of One Health since researchers have achieved one common understanding that One Health is a universal strategy or initiative aimed at increasing multidisciplinary collaboration and communication across all facets of human, animal, and environmental health [19,77,78].

Meanwhile, a new concept named One Welfare is gaining traction. It is founded on the notion of One Health and can be used to address animal welfare and environmental preservation issues. One Welfare, analogous to One Health which is an interface between human, animal, and environmental welfare [79], was developed as an emerging global animal welfare strategy [80]. It acknowledges the relation between animal welfare, human well-being, and environmental sustainability, though it lacks clear and precise definitions at the present. These two concepts have also been extensively debated in veterinary ethics [81], companion animal protection [82], animal sheltering [83], and disease prevention [84], amongst many other domains. Health is fundamental to welfare, and the One Health and One Welfare approaches stress that these terms equally apply to humans and non-humans [85]. That is why the One Health and One Welfare commonly refer to certain subjects or have a high degree of overlap. Given that there is a greater emphasis on the relationship between One Health and the pursuit of fairness and justice in this research, and since the relationship between One Welfare and law is mostly centered on agricultural animals [86], we refer to the emerging strategy as One Health strategy.

We admit that the institutional context of One Health strategy in a particular country can vary and have many different elements–ranging from a matter of epistemological course to cultural anthropology discussions [67,87]. A larger body of social scientists working on the linkage between those elements enhances the understanding of the potential of One Health as a solution to bigger issues such as economic progress and social justice [88,89,90] and reveals that many of these elements are, in fact related to the legal regime in a country [91].

However, literature relating to One Health (or One Welfare) in law is still scarce. According to Phelan, legislation may help address gaps in capacity for preventing, detecting, and responding to new and persistent public health risks, but he places a greater emphasis on international collaboration than on how each country should implement a single health strategy [92]. The pandemics of COVID-19 and other new infectious diseases, as well as the continuing danger of antimicrobial resistance (AMR), have been recognized by the OIE, emphasizing the tight connections between human, animal, and environmental health. As a result, there is an urgent need for a holistic approach (that is, the approach of One Health) to address these issues. Meanwhile, FAO also places a greater focus on the legislative and regulatory implementation of the One Health approach. Indeed, legislation has been proved to be a viable route to One Health [18], but we have not seen a clear map and real evaluation of how the court or legal system would react to the One Health approach for improving animal welfare.

No country is exception to the predicament mentioned above. The application of One Health analytical framework is not new in China now [17], as seen by the Fangcang shelter hospitals during the COVID-19 pandemic [93] as a vivid example. Similar logics have been used in the past to prevent and control the spread of infectious diseases as well as wildlife conservation [94]. For example, in the treatment of the schistosomiasis in the Poyang Lake Basin from 1998 to 1999, scientists from different disciplines formed a scientific research team, use simultaneous intervention to treat susceptible individuals such as humans and cattle, and eventually successfully reduced the morbidity and mortality caused by schistosomiasis [95,96]. However, problems are still there: we cannot see ample literature One Health in law, especially in name of litigation strategy.

Based on support from international organizations, the responsive features embedded in One Health approach to fulminate diseases has garnered considerable attention from academics, the public, and the government. Such features align with the aforementioned principles of legal strategy and strategic litigation–to identify the governing social norm and conceive potential change in the future, which is probably already expressed in some existing legal doctrine [33].

Unlike the theoretical ambiguity, tactically operationalizing One Health in the background of litigation strategy is more explicit, maneuverable and clear. Modern medical law essentially affirms that individuals may be compensated for losses caused by illnesses [97]. “An increase in damages awards to successful plaintiffs tends to increase the filling of legal complaints by increasing the expected value of trial, but it also has an effect in the opposite direction. Potential defendants can often avoid dispute by avoiding the injuries that cause them” [38]. Here compensation may be made for psychological or economic damages [98]. In cases involving zoonotic diseases, this normally entails a mixture of animal health issues and human compensation claims resulting from the illnesses. Even without addressing the theoretical concept of One Health, which is still in its infancy or childhood, we may include environmental protection concerns by splicing and superimposing current litigation strategies. This also reveals pretty high practical applicability of the One Health tactic.

In this study, our core argument is that using One Health litigation strategy to enhance animal welfare is an effective approach. It has been proved that economic analysis can be applied to One Health research since it can quantify outcomes across sectors and make comparisons across sectors and treatments, enabling for the evaluation of policy efficacy across human, animal, and environmental health [48]. To further assess the effectiveness of a litigation strategy statistically, we used two indicators in this study: the lawsuit’s win ratio and the amount of damage award.

**Hypothesis 1** **(H1).** *The use of One Health strategy will be positively associated with higher rates of win in animal health lawsuits.*

We expected the effectiveness of One Health litigation strategy to exist for all aspects of a lawsuit; nevertheless, we expected the magnitude to vary across the judicial process in two predicted methods. Our first proposition builds upon the economic value of One Health eventually being in accordance with court preference. Namely, the court obtains the power to conduct an assessment of the economics of One Health; thereby, the court measurement is initiated by One-Health-related claims. For this reason, we expected increases in damage awards to be a function of the existence of effective One Health litigation strategy in the pleadings and the judgments. Our second prediction is that the extent to which One Health promotion drives judges to protect litigants with One Health claims, however, depends on the amount awarded for damage or compensation decided by the courts. Litigants who utilize litigation strategies themed in One Health in animal health cases could receive more compensation or a higher damage award. We predict that:

**Hypothesis 2** **(H2).** *The use of One Health strategy will be positively associated with higher proportion of damage award in animal health lawsuits.*

## 3. Methodology

### 3.1. Empirical Context

To offer a proper context to test our predictions, we focused on the civil lawsuit sector. Courts in this judicial branch are faced with some of the country’s most severe conflicts, and civil lawsuit conflicts in China are seen as mirrors of social change, where nurtures creative litigation strategies. Regardless of whether laws and regulations are adequate, civil conflicts and cases continue to emerge without waiting for legislators. Many social welfare academia members, holding the viewpoint that the Congress and the administrative bodies are responsible for failures when creating and implementing sufficiently stringent norms in areas such as tobacco control and animal health, have supported litigation as a means of achieving public health policy goals including animal welfare protection [99,100]. Thus, courts in this field need to meet demands from both the social and economic dimensions. For this reason, making judgments requires efficiency in terms of meeting conflict-resolving demands in civil society and, accordingly, the regulatory and normative rules offered by public policies or social welfare goals. As a consequence, we closely examined these judicial cases for court preference for public policies, such as environmental protection and animal welfare, which resulted in significant economic compensation given to litigation participators. Public authorities, especially regulators and policymakers, are willing to respond strategically in this context to support court systems with more mature legislation in the future.

To identify the potential mechanism as we predicted, the context that serves our purpose should be able to (1) isolate One Health related lawsuits from routine civil lawsuits, (2) observe reasons that may persuade judges in the scope of their discretion, and (3) consider the bindings from the legal system which usually appeared in the form of clauses from particular codes in China. If our argument holds in this empirical context, then it would be plausible to conclude that even within an environment with the weakest regulations and norms where no One Health related clauses can be found in the legal system in China. some courts that take the One Health litigation strategy into consideration. Thus, it is rational to assume that since the occurrence of intended judgments are confirmed under the most extreme conditions, it will definitely occur in most settings that value animal health and with higher acceptance of the One Health initiative.

Veterinary scholars have examined One Health-related lawsuits by separating zoonotic from non-zoonotic cases. Waltner-Toews identified the conflicts among observers, practitioners, and scholars in the One Health approach, and stated that facilitating and managing these conflicts should be an important function of the One Health framework [101]. However, he did not attempt to isolate zoonotic cases from health as a whole [102].

Recent developments and changes in the civil lawsuit sector allow this context to satisfy the second benchmark. Since concerns regarding the threat of emerging infectious diseases are growing rapidly, with increases in health costs and enormous efforts directed toward economy stimulation, both courts will not act as policymakers, they are eager to seek conservative solutions. This tendency is reflected both downstream and upstream of the court decision process. While the challenge for animal welfare litigators should be: whether they are able to develop litigation strategies that are safe enough to not touch the boundaries of the judge’s discretion and at the same time gain support in the scope of the judge’s discretion.

### 3.2. Sample and Data

Documenting the effectiveness of litigation strategy themed in One Health is a complicated task; we employed a novel approach to empirically do so: the extent to which courts fulfil demands from the One Health litigation strategy users in civil lawsuit cases. Civil lawsuits reflect that a party has not fulfilled a legal obligation or has not taken timely actions that a prudent animal health protector or practitioner should exercise. Although civil lawsuits cannot measure all difficulties of pursuing the One Health approach, they typically capture substantial disputes related to imprudent action, negligence, mistakes, or mismanagement of the animal–human–environment interface. For example, small enterprises are remiss in animal product invite lawsuits, and veterinarians are remiss in vaccination invite lawsuits.

Our approach involved examining the win ratio and the damage award. Our sample consisted of cases relevant to zoonosis control between 2016 and 2020. Zoonoses are the largest threat to the interface of animal, human, and environmental health. To verify our assumptions, we examined the extent to which claims for One Health, rather than merely animal, human, or environmental health, were supported by the courts. The powers of a judge to order payment of compensation provided us with a method through which to precisely measure the preference of said judges in terms of One Health cases.

The data on civil lawsuit judgments were collected from the China Judgement Online Database, which documents all Chinese civil lawsuit judgments from central to local (federal to district) courts, except in rare circumstances in which judges or settlement terms do not allow judgments to be made available. Although this database allowed us to comprehensively track China civil lawsuit judgments involving particular zoonotic diseases, it does not classify lawsuit judgments by the type of zoonotic disease. As described below, we chose to focus our analysis on certain types of zoonoses that arguably best reflect One Health in practice.

We used two governmental documents to identify the specified animal health cases from 2016 to 2020: (i) List of Zoonotic Diseases, issued and implemented by Announcement No. 1149 of the former Ministry of Agriculture (now the Ministry of Agriculture and Rural Affairs) of the People’s Republic of China on 19 January 2009 and (ii) OIE-Listed Diseases, Infections and Infestations in Force in 2020. We chose five zoonoses under two concerns (i) large scale samples can be observed and collected from China Judgment Online and (ii) there are emerging literature linking animal welfare and One Health to the specified zoonotic disease in China [19,103,104,105,106,107,108]. They are: rabies, highly pathogenic avian influenza, brucellosis, foot and mouth disease and schistosomiasis (here we use the offical English translation of those zoonoses offered in the List of Quarantine Disease for the Animals Imported to the People’s Republic of China).

In the following step, we excluded the cases that were: (i) repeated records, (ii) litigation not linked to animal health violations, and (iii) lacking specific information of the litigants (e.g., incomplete name of the litigation participants). The usable sample consisted of 1520 civil lawsuit judgments from 28 provinces in China. We analyzed the civil lawsuit judgments rather than the lawsuit fillings because fillings may only be associated with mere threats or frivolous legal actions. Thus, we thought that excluding lawsuit fillings from our analysis would be more likely to capture serious occurrences of animal health emergencies. Additionally, off-site or online access to civil lawsuit fillings was not available for the time period of our study.In defining this lawsuit judgment variable for our analysis, we included all judgments, whether the application of One Health litigation strategy was from defendant side or plaintiff side, even though it might have been more convenient for us to envision One Health approach promoters suing defendants to remedy perceived injustices stemming from litigants remiss in promoting the One Health approach.

### 3.3. How to Identify Litigation Strategy Themed in One Health

**Figure 2:**
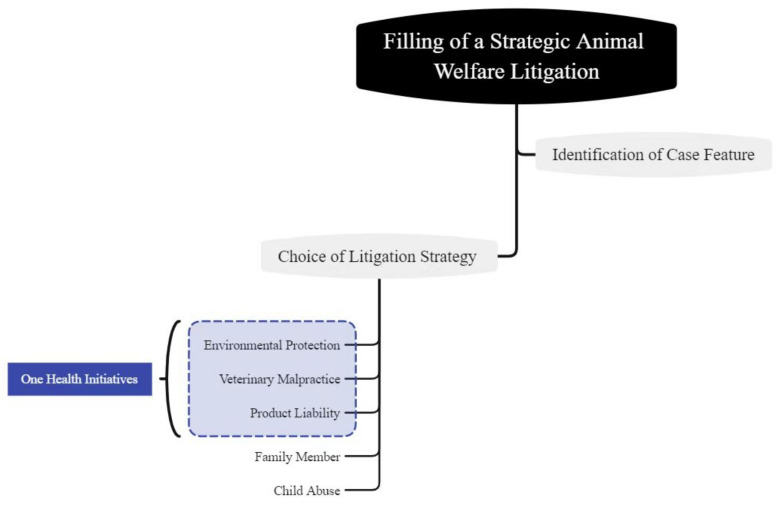
The way to identify a One Health litigation strategy.

Normally, the litigators who start animal welfare litigation will request judicial support holding that an animal has been wrongfully injured, tortured or killed. Judicial support from the courts contains two main elements: (1) a judge’s declaration that animal welfare advocates win; (2) an appropriate method of calculating the monetary damages for compensation for the injury done [42]. As we discussed earlier in this paper, the absence of articles and clauses protecting animal welfare directly from animal law and policy in contemporary China weakens the power of animal welfare advocates’ requests on court. However, animal welfare litigators will not wait in the dilemma, they disassemble the core elements of animal welfare, reorganize them and link them to the interface of human health, animal welfare and environmental sustainability tactically, which fall into the map of One Health or One Welfare, as shown in Figure 2. Here are some examples of One Health litigation strategy.

In the principles of animal law, constant access to fresh, clean drinking water must be secured since it is vital to animal survival. In this sense, freedom from hunger and thirst ranked first among the FAWC’s Five Freedoms [109,110]. In one civil lawsuit judgment from a Wujiang court (see *Xing Chen v. Suzhou Small Animal Protection Association* [111]) determining who is the best carer for animals, the judge considers all the litigants’ economic status, the breeding environment they can offer, and their attitudes toward animal welfare, all of which are evidently important from a One Health perspective:

- …In this case, the plaintiff kept 55 dogs in his home, a building space less than 30 square meters and a poor breeding environment, and these behaviours severely disrupted the regular lives of the neighbours. In addition, none of the dogs had been registered to be kept as pets. The plaintiff reared dogs without permission in violation of Suzhou Dog Management Regulation. As a result, the plaintiff was not allowed to raise dogs. Furthermore, the plaintiff reared 55 dogs and 17 cats in a relatively small room, which had a significant impact on the daily lives of the residents nearby. The court believes that the plaintiff is not appropriate for keeping cats at home either. The Suzhou Small Animal Protection Association is an authorized public welfare organization for protecting homeless animals, it is registered by the Suzhou Civil Affairs Bureau, whereas the plaintiff, is a low-income elder. Therefore, the court believes that the Suzhou Small Animal Protection Association can offer a better environment for the small animals in this case, as well as make it easier for these animals to seek new owners….

The litigation strategy themed in One Health is not privileged to civil cases in China. In one typical criminal case, the prosecutor claimed not only for the preservation of animal welfare, but also for the protection of water quality and the eliminating the possibility that animals suffered by diseases caused by drinking polluted water, which is not a highly litigated issue but hits the overlaps of human health, animal health and environmental protection (See *people v. Xiuyin Yao & Yishuang Zhang* [112]). Thus, we can tell in this case the prosecutor is utilizing One Health litigation strategy.

Among cases relevant to avian influenza control in the real world, we can see the same strategies. In 2020, a Fengtai court in Anhui Province convicted Chuanti Liu and two others illegally covering up and concealing wildlife crime-related income. The prosecutor claimed that the defendants caused damage to the biodiversity and the natural resources. Other than offences against wildlife and the biodiversity, the defendants were also charged for causing interference to the avian influenza control plan schemed by the local government (see *People v. Wang Jinlian et al.* [113]). The litigation strategy from the procuratorate side should be themed in One Health. In another case in 2019, a Renqiu court in Hebei Province not only allowed damages based on loss of poultry farm bankruptcy and high poultry mortality caused by avian influenza, but also on loss of protective value of environmental degradation. Apparently, the plaintiff utilized One Health litigation strategy (See *Mengzeng Bian & Yongli Bian et al. v. Liguo Fan et al.* [114]).

We have to be aware that the litigation strategy themed in One Health is not privileged to the plaintiff side or the lawsuit launchers, a district court in Inner Mongolia Autonomous Region supported the defendant’s answer stating that while the villagers were infected with brucellosis, the water was polluted also and finally ruled that the defendant won (see *Man Zhang v. Wenniute Banner Guangdegong County Balin Avenue Villagers Committee & Li Zhi Yong* [115]).

### 3.4. Dependent Variables

We examined the strategy effectiveness in the decisions of all cases that included the final decision of the winner (*Win*) and the amount awarded to the winner for damage (*Winratio*). We preferred measuring the win ratio of each case, since our hypotheses focused on the effectiveness of the One Health litigation strategy, which is a function of both how the litigants fight with each other in the court and what is echoed from the court. We calculated the variable *Winratio* since the damage awards system set by courts should provide an incentive for animal welfare advocates to take precautions against damage to the human–animal–environmental health interface, thus creating a probable value assessment route. Although consistent with the existing literature, we calculated the variable *Win20* as a baseline for the proportion of the claimed damage awards supported by the courts in the whole amount of damage awards in the claims. We used the common standard, 20%, in most malpractice cases [116].

### 3.5. Independent Variables

#### 3.5.1. Cases Where Litigation Strategy Themed in One Health Can Be Found in Claims from the Plaintiff Side (OA)

We coded this dummy variable OA to identify the characteristics of One Health litigation strategy in civil lawsuit cases where the court made decisions from the plaintiff’s side. OA refers to claims calling for coordination of the human, animal, and environmental health sector (One Health) for the management of zoonoses in the legal documents submitted to the court from the plaintiffs.

#### 3.5.2. Cases Where Litigation Strategy Themed in One Health Can Be Found in Claims from the Defendant Side (AO)

We coded this dummy variable AO to identify the characteristics of One Health litigation strategy in civil lawsuit cases where the court made decisions from the defendant’s side. AO refers to claims calling for coordination of the human, animal, and environmental health sector (One Health) for management of zoonoses in the legal documents submitted to the court from the defendants.

#### 3.5.3. Cases Where No Litigation Strategy Themed in One Health Can Be Found (AA)

We coded this dummy variable AA to identify any characteristics of One Health litigation strategy in civil lawsuit cases where the court made decisions. AA refers to there being no claims calling for coordination of the human, animal, and environmental health sector management of zoonoses in the legal documents submitted to the court from both defendants and plaintiffs.

All the variables used in this paper are listed in Table 2 including the control variables [117,118].

### 3.6. Methodology

We sought to measure how a judge with proper discretion is persuaded by claims embedded with litigation strategy themed in One Health. To test our hypotheses, we used a baseline and the variables of interest. We adopted the Logit and ordinary least squares (OLS) models accordingly [119,120], and for the dummy variables, we used a Logit model [121,122]. The models are specified in Equations (1) and (2):

(1) $$Logit\left(Win\right)=\alpha_{1}+\beta_{1}OA+\beta_{2}AO+\beta_{3}AA+\varphi Controls+\sum Region+\sum Court+\varepsilon ,$$

(2) $$Logit\left(Win20\right)=\alpha_{2}+\beta_{4}OA+\beta_{5}AO+\beta_{6}AA+\phi Controls+\sum Region+\sum Court+\mu ,$$

where *Win* represents a judge’s case decision; *Win20* represents cases with a win ratio higher than 20%; α is the constant; β, ϕ, φ, and ϕ are the regression coefficients; and ε and μ are the stochastic terms.

For the continuous variable *Winratio*, we employed the OLS model (we lost 180 observations where the plaintiffs lost or received no damage awards), represented as follows:

(3) $$Winratio=\gamma +\delta_{1}OA+\delta_{2}AO+\delta_{3}AA+\theta Controls+\sum Region+\sum Court+\sigma ,$$

where *Winratio* denotes the win ratio of the case, γ is the constant, δ and θ are the regression coefficients, and σ is the stochastic term.

## 4. Empirical Analysis

### 4.1. Descriptive Analysis

Table 3, Table 4 and Table 5 present descriptive statistics for the total number of civil judgments of the five focal zoonosis categories (i.e., rabies, avian influenza, brucellosis, aftosa, and bilharziasis), interested variables, and control variables used in this study. Among the final sample of judged cases, 35.99% had claims relevant to the One Health approach with plaintiffs as litigants (referred to as *OA* cases), whereas 14.67% had One Health-related claims with defendants as pursuers (referred to as *AO* cases); the rest of the cases only had claims related to litigants without One Health pursuers (referred to as *AA* cases). Table 3 shows that in our research setting, 88% of the wins went to the plaintiffs, meaning that at least one of the plaintiff’s claims was supported by the court in these cases. As for the percentage of the affirmed damage awards, the average win ratio was 47.6%, and most of the plaintiffs won and received damage awards of between 10% and 50% of their claimed amount (Figure 1. The plaintiffs in 78.1% of the judged cases achieved damage awards with a win ratio higher than 10%, 69.5% with a win ratio higher than 20%, 46.5% with a win ratio higher than 47%, and 44.6% with a win ratio higher than 50%. Table 4 shows that the majority of the judged cases examined in our study were related to rabies, at 61.1%. The rest of the cases were related to avian influenza (21.8%), aftosa (8.6%), brucellosis (5.6%), and bilharziasis (2.7%).

Table 5 illustrates the differences in the mean values between the judged cases grouped by the standard if the win ratio was higher than 10% (win10 = 1 vs. win10 = 0). We found that the average OA and AA in the group (win10 = 0) was lower than that in the group (win10 = 1). Additionally, AO showed an opposite tendency, yet it was the only one that was significant. Table 4 and Table 5 present the differences between the different groups regarding the control variables, including zoonosis type, number of litigants, and the status of the litigants.

Table A1 presents a correlation matrix for which two-tailed tests of significance were used. A positive relationship can be observed between OA and Win (correlation coefficient = 0.095, p<0.05), as well as a negative relationship between AO and Win (correlation coefficient is = −0.255, p<0.05). These findings provide some preliminary evidence in line with H1 and H2, meaning compared with cases where no litigation strategy themed in One Health can be found, that litigators equipped with litigation strategy themed in One Health were more likely to win. In addition, to avoid potential multicollinearity between variables, we tested for the variance inflation factors (VIFs) of all of the variables, excluding the dummy variables, which ranged from 1.06 to 3.08, well below the cut-off value of 10, indicating that multicollinearity was not a major concern in this study.

### 4.2. Main Findings

Table A2 presents the empirical results from the regression models. We report findings from nine panel models, and the dependent variables are *Win*, *Win20*, and *Winratio*. Models 1, 4, and 7 contained only independent variables to test the effects of the litigant’s characteristics related to the One Health approach on the judgment of wining and the amount of damage awards received. Models 2, 5, and 8 incorporated the plaintiff’s characteristics, including the number of plaintiffs and the status of the plaintiffs. Models 3, 6, and 9 included all of the independent variables and all of the control variables. All the models were controlled by two-way fixed effects: the region and the level of court.

Models 1–3 show the regression results with *Win* as one dependent variable. As predicted in Hypothesis 1, that litigation strategies themed in One health are proved to be effective in animal health lawsuits. Our results in these models supported our hypothesis. Model 3 revealed a positive and significant coefficient of *OA* on the probability to win (β=0.564, p<0.05), indicating that compared with litigators who do not utilize the litigation strategy themed in One Health, plaintiffs equipped with litigation strategy themed in One Health have a higher probability of winning. Thus, Hypothesis 1 is supported. Conversely, we found negative and significant coefficients of *AA* on their probability to win in the three models (e.g., in Model 3: β=−1.368, p<0.01). This implied that compared to civil lawsuit cases without any One Health pursuers and bargainers as litigants, cases with no One-Health-related litigants as plaintiffs against defendants with One Health pursuits are associated with a lower probability of winning, thereby supporting Hypothesis 1.

Models 4–6 present the results of the regressions on *Win20*, analyzing the cases where the *winratio* of plaintiffs is above 20%. We hypothesized in Hypothesis 2 that litigators equipped with litigation strategy themed in One Health enjoy advantages in the settlement of damage awards. The coefficients of *OA* were positive and significant in three models (e.g., in Model 6: β=0.5, p<0.01). These findings are consistent with Hypothesis 2, implying that in the case where a One Health litigation strategy utilizer acts as the plaintiff against defendants without any litigation strategy themed in One Health, they are more likely to get a *winratio* above 20% in contrast to cases with no One Health-related litigants. In addition, we found that the coefficients of *AO* were negative and significant in three models (e.g., in Model 6: β=−1.061, p<0.01). This implied that once the plaintiffs start the lawsuits without being equipped with litigation strategies themed in One Health whereas the defendants utilize those strategies to defend themselves in courts, the plaintiffs are less likely to obtain a winratio above 20% in comparison to cases where no litigation strategies themed in One Health can be found. Thus, Hypothesis 2 is supported.

Models 7–9 show the regression results with *Winratio* as the dependent variable, which is calculated as the ratio of the court-granted damage compensation awards to the amount claimed by the plaintiffs. In Model 9, the coefficient of *OA* was positive and significant (Model 9: β=0.056, p<0.01). The results provide further evidence to support Hypothesis 2, meaning that in cases where a litigator equipped with litigation strategy themed in One Health sues someone without any litigation strategies themed in One Health, they are more likely to achieve a higher win ratio compared with cases where no litigation strategy themed in One Health can be found. In addition, the coefficients of AO remained negative and significant in the three models (e.g., in Model 9: β=−0.159, p<0.01), which indicated that when a defendant utilizes litigation strategy themed in One Health to fight against an ordinary litigant without One Health related answers, the case is less likely to achieve a high win ratio compared to cases where no litigation strategy themed in One Health can be found. These results further support Hypothesis 2.

### 4.3. Alternative Explanation and Robustness Tests

We conducted additional tests to check the robustness of the results. First, we adopted probit models to test Hypothesis 1; the results in all of the models are consistent with our findings. Second, we reran the probit models using *Win50* as the dependent variable instead of *Win20*; the results were more profound than before, and they are consistent with our hypotheses. Third, we measured *Winratio* by taking the logarithm form and rerunning our main models separately for each additional independent variable. Our results concerning the damage awards agree with the findings from the previous models. The results of these models are not provided here, but they are available upon request.

## 5. Discussion

Overall, at least three contributions emerge here. To begin with, this study draws on prior research to address an animal law issue with significant public policy implications [123]. To sum up, we have discovered that, in absence of direct and powerful written rules, using One Health litigation strategy effectively increases the probability of winning animal health cases on court, which might signal the potential of positive implications for animal welfare. For policymakers, we believe that animal law should be strengthened to make it more compatible with One Health strategy mentioned here. This will streamline and eliminate obstacles to animal welfare protection. For animal welfare advocates who are considering filing to the court, we recommend that they assess whether judges in their jurisdictions accept One Health initiatives and, if feasible, aggressively and properly utilize One Health litigation strategy. The suitable circumstances can be measured by conducting a legal research on officially recorded cases. Although veterinary public health research on One Health has had little to do with law and much less with litigation strategy, the importance of this study is apparent in terms of public policy protecting animal welfare. In particular, this research focuses on and extends litigation strategies in the field of animal protection, giving specific emphasis to their importance, dynamic changes, and court debating methodologies.

Second, we provide an analytical review of animal welfare litigation strategies, in response to Smith, Fernandez and Staker’s claim that successful animal welfare litigation strategies may decrease barriers to animal protection at the societal level and promote consensus on animal welfare protection [124,125,126]. By summarizing and categorizing the effective litigation strategies that existed in China, we generated a toolbox enabling litigators to combine, modify, and develop new litigation strategies based on the specifics of animal welfare that needs to be protected. In this regard, we concur with Aldana, Osburn and Radeski’s studies, suggesting that an urgent and strategic utilizing One Health strategy would improve wildlife and ecosystem health, thus promoting animal welfare [127,128,129]. What makes this research unique is that we revealed the mechanism of promoting animal welfare from a law and economics perspective [130,131].

Finally, we empirically verified our arguments by analyzing a cross-sectional dataset that spans five years and contains five zoonoses-related cases dispersed across 29 provinces. It is a first-of-its-kind attempt to statistically assess the efficacy of one particular litigation strategy in animal law. However we are not that willing to stop here. Our research weighs anchor from the perspective of law and economics with an emphasis on animal health and a remote destination in futural animal welfare protection. The majority of prior research discussing the relevance of economics to the study of animal welfare was consumer-focused, debating the costs and advantages of animal welfare promotion [132]. Here it was empirically and statistically proven that using One Health litigation strategy results in higher win rates and higher proportions of damage compensation awards. Both the plaintiff side and the defendant side may pay more attention to animal health issues due to the high winning rate and high compensation amount which is usually regarded as some kind of economic loss, and may begin to improve animals’ living conditions, surrounding environment and promote animal welfare gradually and finally.

In terms of study limitations, the potential impact on animal welfare caused by using litigation strategy themed in One Health has not yet been statistically and clearly verified. In future research, it will be worth to explore the mediating effect of animal health on the relationship between One health strategy and animal welfare in detail. For the second point, our analysis does not take into account those recent changes in animal health policies in China (i.e., the Congress passed and enacted the revision of Animal Disease Control Law in 2021). Alternatively, we have not yet assessed the effect on the application of animal welfare litigation strategies of a potential renewal of the One Health concept after the 2020 COVID-19 pandemic. Considering China’s long time isolation and blockade in response to the pandemic, new case admission in court system had been halted at that time period, and many potential animal protection claims have been postponed or shelved. This is an important challenge that future research may need to overcome. For the second point, animal law and policy in China are at the early stage, the litigation strategies themed in One Health may not be duplicated in other countries directly when dealing with animal health issues, though we would like to contribute to enriching the literature of global animal health law. In future research, it will be important to examine how litigation strategies themed in One Health can be used in countries with different legal traditions and judicial systems. Furthermore, more systematic research in this area such as qualitative analysis of single cases may be worthy of exploration. The policy implications of cases containing One Health related litigation strategies for those different of categories of animal welfare advocates might be different, which necessitates further in-depth research.

## 6. Conclusions

This article is among the first to provide comprehensive evidence of the impact of utilizing the litigation strategy themed in One Health as well as of the litigation strategy toolbox for animal welfare advocates’ attempts to protect animals. Contributing to an application-based view of legal strategy, we have mapped out how litigation strategies themed in One Health can stimulate animal health in courts in contemporary China, particularly in zoonosis-related cases. In addition, it povided an explicit explanation for why this litigation strategy has the potential to improve animal welfare as a whole.

## Figures and Tables

**Table 1:** Strategic litigations for animal welfare and responses from the court system.

Strategy	Definition	Examples in Current Literature Worldwide	Symbolic Judgments in China
Environmental protection	Applying creative tactics such as filing suit under environmental laws (The Clean Water Act, Clean Air Act, the Migratory Bird Treaty, and the National Environmental Policy Act) may result in some benefits in the war against animal cruelty [39].	To preserve the well-being of wildlife, filling lawsuit to set precedents for the preservation of biodiversity; to protect the animals adjacent to pollution sites, filling lawsuit to set precedents for the control of contaminated sites.	In one case brought to court by an environmental NGO (Friends of Nature), the plaintiff claimed that the construction of one hydro-power will ruin the core habitat of an endangered green peacock and asked the court to stop the project. The request was finally approved [40].
Child abuse	To prevent child abuse, one choice is to prevent animal abuse for animal abuse signals as a predictor of violence against people in the future.	Petition to the court to uphold a ban on children watching animal cruelty-related videos products and affirm that any animal cruelty-related behavior should be prohibited in a further step.	A Jingzhou district court in Hubei Province ruled that cat abuse videos will cause harm to student watchers’ mental health, so the cat abuse behavior should be banned (See *Li Qin v. Fan Yuanqing* [41]).
Vet malpractice	Michigan has a specific provision requiring the providing of veterinarian care: Adequate care means the provision of sufficient food, water, shelter, sanitary conditions, exercise, and veterinary medical attention in order to maintain an animal in a state of good health. Mich. C.L.A. Section 750.50(1)(a) [42].	Petition to the court to affirm that the veterinarian is responsible for providing enough water, food, and a sanitary environment, etc. for animals.	A Shanghai appellate court ruled that the plaintiff need to provide evidence strong enough for the certified vet’s treatment to the pet cat. However the plaintiff didn’t and failed (*Chaopeng Zhong v. Shanghai Duoqu Companion Animal Co. Ltd.* [43]).
Product liability	Threats to animal welfare, including stress-induced immunosuppression and promotion of foodborne pathogens, and genetic modifications have the potential to compromise the healthiness and safety of food [44].	Petition to the court to affirm that there is a link between the failure to meet the farm animal welfare requirements and the failure to meet the food quality standards	A Hangzhou appellate court ruled that animal products from regions where African Swine Fever and Foot and Mouth Disease outbreak should not be imported and transacted (*Hongmei Chen v. Dongning County Russian Commodity Shop* [45]).
Quasi-family member	Judges need to be mindful of the importance of pets as family members in their decisions in juvenile and family law matters. The animal owner cannot recover damages for the loss of companionship he/she suffered.	Petition to the court to support the compensation request for the loss of mutual companionship due to the separation of pets and their owners.	A Shanghai district court ruled that the plaintiff has been in the companionship with his pet dog for several years and they are living like family members to each other, the loss of the companionship should be considered when deciding the mental damage (*Jun Li v. Shanghai Peishi Animal Clinic Co. Ltd.* [46]).

**Table 2:** Variables and their definitions.

Variable	Definition
**Dependent variables**	
Win	Dummy = 1 if at least one of the plaintiff’s claim(s) is affirmed by the court
Winratio	Ratio of the amount of damage awards granted by the court to the amount claimed by the plaintiff
Win20	Dummy = 1 if the *Winratio* is higher than 20%
**Independent variables**	
OA	Dummy = 1 when the litigation strategy themed in One Health can be found in the plaintiff’s claim
AO	Dummy = 1 when the litigation strategy themed in One Health can be found in the defendant’s claim
AA	Dummy = 1 when no litigation strategy themed in One Health can be found in the whole set of the legal document for this case
**Control variables**	
A. Characteristics of the cases	
*Zoonotic feature*	Zoonoses dealt with in the judgment
Court	Dummy variables of the courts
Region	Region where the court is located
B. Characteristics of the plaintiffs and defendants	
Number	Number of plaintiffs and defendants
*Plaintiff ownership*	Dummy = 1 when the plaintiffs are state owned enterprises/private firms
*Defendant ownership*	Dummy = 1 when the defendants are individuals/state-owned enterprises/private firms

**Table 3:** Descriptive statistics of the dependent variables.

Variable	*N*	Mean	SD	Min.	Max.
Win	1520	0.884	0.320	0	1
Win10	1520	0.781	0.413	0	1
Win20	1520	0.695	0.460	0	1
Win47	1520	0.465	0.498	0	1
Win50	1520	0.446	0.497	0	1
Winratio	1520	0.476	0.360	0	1

**Table 4:** Descriptive statistics for the independent and control variables.

Variable	*N*	Mean	SD	Min.	Max.
OA	1520	0.359	0.480	0	1
AO	1520	0.147	0.354	0	1
AA	1520	0.492	0.500	0	1
Zoonosis type (rabies)	1520	0.611	0.487	0	1
Zoonosis type (avian influenza)	1520	0.218	0.413	0	1
Zoonosis type (brucellosis)	1520	0.056	0.231	0	1
Zoonosis type (aftosa)	1520	0.086	0.280	0	1
Zoonosis type (bilharziasis)	1520	0.027	0.163	0	7
PE	1520	1.088	0.434	0	20
DE	1520	1.320	0.843	0	1
State DE	1520	0.068	0.252	0	1
Corporate DE	1520	0.238	0.426	0	1
Individual DE	1520	0.777	0.416	0	1
State PE	1520	0.015	0.124	0	1
Corporate PE	1520	0.121	0.326	0	1

DE and PE here means the nature of the defendants’ ownership and plaintiff’s ownership.

**Table 5:** Descriptive statistics of the judged case observations grouped by *win10*.

	*win10* = 0 (*N* = 333)				*win10* = 1 (*N* = 1187)			
	Mean	SD	Min.	Max.	Mean	SD	Min.	Max.
OA	0.294	0.456	0	1	0.378	0.485	0	1
AO	0.279	0.449	0	1	0.110 *	0.313	0	1
AA	0.426	0.495	0	1	0.511	0.500	0	1
Zoonosis Disease								
Rabies	0.522	0.500	0	1	0.636 *	0.481	0	1
Avian influenza	0.228	0.420	0	1	0.215 *	0.411	0	1
Brucellosis	0.690	0.253	0	1	0.053 *	0.224	0	1
Aftosa	0.147	0.354	0	1	0.069 *	0.253	0	1
Bilharziasis	0.033	0.178	0	1	0.026 *	0.159	0	1
PE	1.084	0.378	1	5	1.089	0.448	0	7
DE	1.285	0.828	1	9	1.330 *	0.847	0	20
State DE	0.087	0.282	0	1	0.063	0.243	0	1
Corporate DE	0.366	0.482	0	1	0.202	0.402	0	1
Individual DE	0.660	0.474	0	1	0.810 *	0.392	0	1
State PE	0.006	0.077	0	1	0.018	0.135	0	1
Corporate PE	0.930	0.291	0	1	0.129 *	0.335	0	1

Significance is based on the group of Win: Mann–Whitney U-test for dummy variables and *t*-test for continuous variables. * p<0.05.

## Data Availability

The datasets generated and/or analysed during the current study are available from the corresponding author upon reasonable request.

## Appendix A. Correlation Matrix (Table A1)

**Table A1:** Correlation matrix.

	Win	Win20	Win Ratio	OA	AO	AA	Rabies	Avian Influenza	Brucellosis	Aftosa	Bilharziasis	PE	DE	State DE	Corporate DE	Individual DE	State PE	Corporate PE
Win	1																	
Win20	0.536 *	1																
Win ratio	0.542 *	0.800 *	1															
OA	0.095 *	0.085 *	0.124 *	1														
AO	−0.255 *	−0.131 *	−0.065 *	−0.311 *	1													
AA	0.089 *	0.010 *	−0.073 *	−0.738 *	−0.410 *	1												
Rabies	0.138 *	0.001	−0.206 *	−0.287 *	−0.313 *	0.497 *	1											
Avian influenza	−0.037	0.059 *	0.239 *	0.056 *	0.324 *	−0.284 *	−0.664 *	1										
Brucellosis	−0.062 *	−0.010	−0.014	0.214 *	0.042	−0.236 *	−0.308 *	−0.129 *	1									
Aftosa	−0.133 *	−0.069 *	0.022	0.160 *	0.033	−0.177 *	−0.383 *	−0.161	−0.074 *	1								
Bilharziasis	−0.001	−0.018	−0.007	0.133 *	−0.002 *	−0.126 *	−0.212 *	−0.089 *	−0.041	−0.089 *	1							
PE	0.020	−0.0004	−0.020	0.072 *	0.031	−0.091 *	−0.029	−0.035	0.023	−0.035	0.202 *	1						
DE	0.016	0.085 *	0.044	−0.068 *	0.104 *	−0.008	−0.021	0.008	−0.021	−0.010	0.049	0.086	1					
State DE	−0.064 *	−0.052 *	−0.050 *	0.166 *	0.107 *	−0.236 *	−0.121 *	0.014	0.147 *	−0.017	0.145 *	0.209 *	0.1004 *	1				
Corporate DE	−0.072 *	−0.091 *	−0.024	0.196 *	0.115 *	−0.270 *	−0.297 *	0.104 *	0.130 *	0.184 *	0.122 *	−0.0444	0.059	−0.047	1			
Individual DE	0.132 *	0.101 *	0.034	−0.256 *	−0.116 *	0.328 *	0.318 *	−0.119 *	−0.197 *	−0.137 *	−0.131 *	−0.037	0.132 *	−0.347 *	−0.703 *	1		
State PE	0.012	0.038	0.090 *	0.059 *	0.051 *	−0.093 *	−0.116 *	0.073 *	0.060 *	0.037	0.010	0.012	0.036	0.007	0.028	−0.021	1	
Corporate PE	−0.035	0.088 *	0.206 *	−0.046	0.266 *	−0.144 *	−0.351 *	0.292	0.092 *	0.132 *	−0.050 *	−0.057 *	0.096 *	−0.044	0.118 *	−0.111 *	−0.030	1

Standard errors are reported in parentheses. * p<0.10.

## Appendix B. Empirical Findings from the Logit Estimations and Ordinary Least Squares (OLS) (Table A2)

**Table A2:** Empirical findings from the Logit estimations and ordinary least squares (OLS) estimations.

	Win			Win20			Winratio		
	(1)	(2)	(3)	(4)	(5)	(6)	(7)	(8)	(9)
OA	0.353	0.412	0.564 **	0.296 **	0.359 **	0.500 ***	0.028	0.040 *	0.056 ***
	(0.250)	(0.259)	(0.286)	(0.150)	(0.155)	(0.164)	(0.021)	(0.020)	(0.021)
AO	−1.349 ***	−1.434 ***	−1.368 ***	−0.945 ***	−1.129 ***	−1.061 ***	−0.147 ***	−0.173 ***	−0.159 ***
	(0.257)	(0.267)	(0.288)	(0.188)	(0.200)	(0.211)	(0.031)	(0.030)	(0.031)
AA	Baseline	Baseline	Baseline	Baseline	Baseline	Baseline	Baseline	Baseline	Baseline
Zoonosis type (avian influenza)	1.498 ***	1.541 ***	1.539 ***	0.946 ***	0.992 ***	0.999 ***	0.162 ***	0.165 ***	0.162 ***
	(0.315)	(0.319)	(0.324)	(0.236)	(0.247)	(0.248)	(0.041)	(0.041)	(0.040)
Zoonosis type (brucellosis)	0.331	0.282	0.413	0.018	−0.063	0.041	−0.093	−0.096*	−0.081
	(0.315)	(0.319)	(0.324)	(0.236)	(0.247)	(0.248)	(0.041)	(0.041)	(0.040)
Zoonosis type (bilharziasis)	1.649 ***	1.607 ***	1.893 ***	−0.027	0.138	0.297	−0.068	−0.030	−0.013
	(0.721)	(0.733)	(0.761)	(0.449)	(0.470)	(0.472)	(0.073)	(0.074)	(0.073)
Zoonosis type (rabies)	1.636 ***	1.835 ***	1.749 ***	0.306	0.575 **	0.484 **	−0.138 ***	−0.077 **	−0.090 **
	(0.287)	(0.307)	(0.307)	(0.213)	(0.229)	(0.231)	(0.038)	(0.039)	(0.039)
Zoonosis type (aftosa)	Baseline	Baseline	Baseline	Baseline	Baseline	Baseline	Baseline	Baseline	Baseline
State DE			0.372			−0.412			−0.060
			(0.424)			(0.308)			(0.047)
Corporate DE			0.846 **			−0.248			−0.023
			(0.385)			(0.237)			(0.032)
Individual DE			1.374 ***			0.297			0.040
			(0.405)			(0.258)			(0.036)
State PL		0.985	0.941		1.227 **	1.077 *		0.280 ***	0.252 ***
		(0.797)	(0.823)		(0.566)	(0.567)		(0.072)	(0.069)
Corporate PL		0.650 **	0.647 **		1.194 ***	1.197 ***		0.213 ***	0.210 ***
		(0.288)	(0.284)		(0.228)	(0.230)		(0.032)	(0.032)
Number		PL	PL, DE		PL	PL, DE		PL	PL, DE
Region	Controlled	Controlled	Controlled	Controlled	Controlled	Controlled	Controlled	Controlled	Controlled
Court	Controlled	Controlled	Controlled	Controlled	Controlled	Controlled	Controlled	Controlled	Controlled
Constant	1.321 **	0.680	−0.579	0.494	0.220	−0.219	0.403 **	0.396 ***	0.383 ***
	(0.577)	(0.661)	(0.777)	(0.335)	(0.388)	(0.453)	(0.080)	(0.084)	(0.092)
Prob > $\chi^{2}$; Prob > *F*	0	0	0	0	0	0	0	0	0
Pseudo $R^{2}$	(0.1357)	(0.1454)	(0.1597)	(0.0654)	(0.0835)	(0.0950)	(0.1437)	(0.1786)	(0.1862)
Obs.	1499	1499	1499	1501	1501	1501	1503	1503	1503

Standard errors are reported in parenthesis. * p<0.10; ** p<0.05; *** p<0.01. PL here means the nature of the plaintiff’s ownership.
